# Bisphosphonate-coated external fixation pins appear similar to hydroxyapatite-coated pins in the tibial metaphysis and to uncoated pins in the shaft: A randomized trial

**DOI:** 10.3109/17453674.2013.797315

**Published:** 2013-05-31

**Authors:** Sören Toksvig-Larsen, Per Aspenberg

**Affiliations:** ^1^Department of Orthopedics, Hässleholm Hospital, Hässleholm, and Department of Orthopedics, Clinical Sciences, Lund University, Lund; ^2^Orthopedics, Clinical and Experimental Medicine, Faculty of Health Sciences, Linköping University, Linköping, Sweden.

## Abstract

**Background and purpose:**

External fixation pins tend to loosen with time, especially from cancellous bone. A coating that releases a bisphosphonate has been shown to improve the fixation of dental implants in humans. We now tested a bisphosphonate coating on steel pins for external fixation. The primary hypothesis was that coated pins would be better fixed in the diaphysis.

**Methods:**

20 patients with medial knee osteoarthritis underwent proximal tibial correction osteotomy with hemicallotasis. They received a pair of pins (Orthofix) in the tibial shaft, one bisphosphonate-coated and one uncoated. Another pair of pins was inserted in the metaphysis, near the joint. This pair was a bisphosphonate-coated pin and an HA-coated pin. All pins were inserted according to a random list. The pins were removed after the osteotomy had healed (8–15 weeks), and extraction torque served as the predetermined main outcome variable.

**Results:**

No pins showed clinical signs of loosening. Removal torque for the shaft pins was 6.6 Nm (SD 2.2) for the bisphosphonate and 6.0 Nm (SD 2.5) for the uncoated (difference = 0.5, 95% CI: –0.03 to 1.3). Removal torque for the metaphyseal pins was 4.4 Nm (SD 1.3) for the bisphosphonate-coated and 4.2 Nm (SD 1.6) for the HA-coated (difference = 0.2, 95% CI: –0.5 to 1.0).

**Interpretation:**

We could not show any improved cortical fixation, but the metaphyseal findings are striking. In a previous study on 19 patients with a similar layout, HA-coated and uncoated pins were compared. In the metaphysis, all 19 uncoated pins loosened before removal. It was concluded that uncoated pins could not be used in the metaphyseal region. The present results suggest that a bisphosphonate coating enables metaphyseal fixation similar to that of hydroxyapatite coatings, with no difference from uncoated pins in cortical bone.

External fixation with threaded pins is an easy and practical method for minimally invasive fracture fixation. However, it is mainly used only for temporary fixation, because of problems with loosening and pin-tract infection. This problem can be largely overcome by the use of pins with a hydroxyapatite coating (Magyar et al. 1997, Pommer et al. 2002). However, hydroxyapatite works by enabling bone formation directly on its surface, creating a chemical bond between the crystals of the coating and those of the newly-formed bone. This can make the screws hard to remove (Sanden et al. 2000). Screws are designed to convert mechanical shearing forces into compression: a nail resists shearing force when one tries to push or pull it, but screws resist compression forces at their threads. Therefore, the strength of screw fixation is not so much dependent on osseointegration as on the strength of the bone that surrounds the threads. A hydroxyapatite coating only exerts surface effects: it does not strengthen the surrounding bone. When a hydroxyapatite screw becomes osseointegrated, the newly-formed bone layer on its surface will transmit forces to the surrounding bone more efficiently, which may then lead to improved bone strength by adaptation. In contrast, a bisphosphonate released from the surface will influence the surrounding bone directly, by changing the balance between bone formation and resorption. It is therefore possible, at least in theory, to make a screw that will more quickly be able to resist compression forces and still be easy to remove.

Bisphosphonate coatings can be made by applying a nanometer-thick protein layer to the implant surface, to which a bisphosphonate can be made to adhere (Tengvall et al. 2004). Such coatings have been tested in a series of rat experiments with steel or titanium screws. The results showed a rapid improvement in fixation in cancellous and cortical bone (Wermelin et al. 2007). In cancellous bone, this was achieved by the creation of a dense bone envelope around the screw, with newly-formed bony threads that resist pullout forces (Wermelin et al. 2008). However, removal torque was also increased, probably because a larger part of the implant surface is in contact with bone. The mechanism of the bisphosphonate effect in cortical bone has not been fully determined, but it has been shown in previous experiments that implants in cortical bone lose part of their fixation due to postoperative resorption at the interface (Dhert et al. 1998). This resorption might be inhibited by the bisphosphonate, but we have also seen thickening of the cortex at the insertion site.

Following these experiments, a clinical trial has been performed, showing that dental implants become better fixed if they are coated with a bisphosphonate (Abtahi et al. 2012a). We now hypothesized that threaded pins for external fixation might also benefit from a bisphosphonate coating, and we tested this in patients undergoing hemicallotasis.

Hemicallotasis is a method for valgus or varus osteotomy in cases of osteoarthritis of the knee. It uses an external fixation device with 2 threaded pins in the tibial shaft and 2 in the metaphyseal bone, quite near the tibial joint surface (Magyar et al. 1998). We normally use uncoated pins for the shaft and hydroxyapatite-coated pins for the metaphysis, as uncoated pins have a strong tendency to loosen at this cancellous bone site (Magyar et al. 1997).

In this randomized trial, we compared bisphosphonate-coated pins with uncoated ones in the shaft, and with hydroxyapatite-coated pins in the metaphysis. The primary hypothesis—further specified below—was that the coating would improve fixation, as measured by removal torque.

## Patients and methods

### Overview

20 patients, who underwent hemicallotasis, received a pair of pins in the shaft and another pair in the metaphysis. At each location, 1 pin was coated with bisphosphonate. In the shaft the control pin was uncoated, and in the metaphysis it was coated with hydroxyapatite. Placement was randomized. It was blinded in the shaft, but not in the metaphysis. Evaluation was based on removal torque after the osteotomy had healed. The study was performed at Hässleholm Hospital, Hässleholm, Sweden, and registered at clinicaltrials.gov (PFX2009378).

The study complied with the Helsinki Declaration, and ethical approval was granted at Etikprövningsnämnden Lund (where the protocol is available, no. 2009/378). The first patient was operated on May 11, 2011 and the last on March 8, 2012.

### Implant preparation

We coated Orthofix standard conical external fixation pins pins (5/6 mm; Orthofix, Bussolengo, Italy): 110/40 mm pins (n = 20) for the shaft, and 130/50 mm pins for the metaphysis (n = 20). Coating was performed as follows.

The screws were cleaned in 2 vol% Triton X with 2 vol% acetic acid by ultrasonication for 5 min, rinsed in MilliQ water, ultrasonicated for 5 min in 70 vol% ethanol, and then rinsed thoroughly. Thereafter, the pins were etched with 100 mg/mL Fe(III)Cl in 24% HCl for 30 min. After etching, the pins were ultrasonicated in 2 vol% acetic acid for 5 min, and then in MilliQ water for 5 min. Thereafter, they were dried in N2 and kept in sealed tubes before coating.

The threaded part of the pins was coated with a fibrinogen multilayer incorporating 400 ng/cm^2^ of zoledronic acid in a process modified from Tengvall et al. (2004). Briefly, the screws were incubated first in 2 mg/mL medical-grade fibrinogen (Haemocomplettan; CSL Behring, Germany) for 10 min, and then in 0.5 µg/mL zoledronic acid for 30 min. After drying and single packaging in double peel-pouches, all screws were sterilized with γ-irradiation at 17–25 kGy (Beta-Gamma Service, Wiehl, Germany)

The 20 hydroxyapatite pins were similar in design and size, except for the coating (Orthofix OsteoTite).

### Randomization

The order in which the patients were to receive a bisphosphonate-coated pin distally or proximally in the shaft was decided by shuffling sealed envelopes in blocks. Then they were opened, and a random list was produced. The pin bags, each containing 1 pin, were then labeled with consecutive patient numbers, and “D” for distal or “P” for proximal. The surgeon and all the clinical staff were blinded regarding treatment. It is not possible to discern the bisphosphonate-coated pins from the uncoated pins by visual inspection.

The pins for the metaphysis (coated with bisphosphonate or hydroxyapatite) look slightly different, and this part of the study could not be blinded with any certainty. Numbered, sealed envelopes were prepared as above and opened at surgery, telling the surgeon which pin should be inserted laterally or medially.

### Patients

The power analysis was based on a previous study of similar design (Toksvig-Larsen and W-Dahl 2008). In that study, the variation (SD) of the difference between insertion and extraction torque was 1.75 N. We therefore assumed a random variation of this difference between 2 pins to be 1.75 × 20.5. We regarded a difference in torque between 2 screws of 1 N as clinically important, which meant that 19 patients would be needed in order to obtain a power of 90%.

20 patients on the waiting list for high tibial osteotomy for medial or lateral osteoarthritis, using the hemicallotasis technique, were invited to participate in the study after screening, and all accepted. There were no exclusion criteria other than the clinical criteria for not using hemicallotasis.

Surgery was performed as previously described (Toksvig-Larsen and W-Dahl 2008) using the Orthofix T-garche as external fixator. 4 pins, 2 HA-coated in the metaphyseal bone and 2 non-coated in the diaphyseal bone, were inserted extraarticularly. A 5-cm longitudinal skin incision was made at the distal level of the tuberosity, and was then tested with regard to the extension of the osteotomy, which was judged to be sufficient if the gap could easily be opened 4–5 mm. For valgus deformity, the surgical procedure was the same except that a fibulotomy was performed 10–15 cm below the head of the fibula. The patients were allowed free mobilization and full weight bearing after the operation. Most patients were discharged on the day of surgery.

The distraction started 14 days postoperatively. 8 weeks postoperatively, the fixation was dynamized to stimulate bone healing. At 8–10 weeks postoperatively, depending on symptoms, a bone-healing control was done by radiography and ultrasound. If these had satisfactory findings, the external frame was removed and the patient tested full weight bearing for 1 h. When this was pain-free, the pins were removed. If the patient developed symptoms during the test, the fixator was used for 2 additional weeks.

Insertion and extraction torque was measured with a torque force screwdriver (range 0–11 Nm; Orthofix, Bussolengo, Italy) (Toksvig-Larsen and W-Dahl 2008). Extraction of the pins was performed by a trained nurse who was unaware of the pin treatment. The pins were always removed in the same order, and the patients were asked to rate (verbally) the extraction pain on a scale from 0 to 10 (none to worst possible).

### Wound care and monitoring

A study nurse performed pin-site care once a week (Toksvig-Larsen and W-Dahl 2008), checking for possible pin-track problems and pain. Signs of pin-track infection were graded according to the Checketts-Otterburn scale from 0 to 6, where 0 means no signs, 1 means redness and minimal discharge, and 5 means involvement of the bone so that fixation is affected (Checketts et al. 2000).

### Hypotheses, criteria for premature termination, and statistics

The primary hypothesis was that removal torque minus insertion torque in the shaft would show a higher value for the bisphosphonate-coated pins than for the uncoated pins. There were 2 secondary hypotheses: that the metaphyseal bisphosphonate-coated pins would not become loose (i.e. produce unmeasurable torque resistance), and that shaft pins could be removed without any problems.

Because uncoated pins are known to carry a high risk of loosening in the metaphysis, the protocol stipulated that if 4–6 patients had loosening of a bisphosphonate-coated pin, the metaphyseal part of the study should be terminated and the remaining patients should have both of their metaphyseal pins hydroxyapatite-coated.

Removal torque values were compared using paired t-test (SPSS Statistics 20). Confidence intervals (CIs) used an α of 0.05. Pain estimations were compared using Wilcoxon’s test for paired data.

Because of conflicts of interests, PA had no contact with the patients and had no communication with the other author regarding the performance of the study, between study start and data lock.

## Results

### Overall results

The pins were removed after 8–15 weeks (mean 12, SD 1.8). Insertion and removal torque was registered for all pins. There was no case of clinical pin loosening. When not otherwise specified below, the Checketts-Otterburn grade was 0 or 1. Estimation of pain at removal was not performed in 3 patients.

### Shaft pins

Wound inspection showed no difference between the 2 pins. 2 patients had infection of both pins (Checkett-Otterburn grade 2). The infections resolved within a week. Estimation of pain at removal was median 6 for both types of pins (difference = 0 units, range: 6 to –4). In 1 patient, low values were recorded for both pins (0 Nm and 1 Nm). However, the patient had a constant Checketts-Otterburn grade of 1 for all pins, and minimum recorded use of analgesics. Because pin loosening is normally associated with a higher Checketts-Otterburn grade (swelling, secretion) and pain, we do not regard this as a case of loosening: possibly the torque values were incorrect. The values have not been excluded.

The difference between diaphyseal removal and insertion torque was 0.28 Nm (SD 1.9) for the bisphosphonate pins (meaning a slight improvement after insertion), whereas it was -0.15 Nm (SD 2.1) for the uncoated pins (difference = 0.42, CI: –0.28 to 1.1).

Removal torque for the shaft pins was 6.6 Nm (SD 2.2) for the bisphosphonate and 6.0 Nm (SD 2.5) for the uncoated (difference = 0.5; CI: –0.03 to 1.3).

### Metaphyseal pins

Wound inspection showed no difference between the 2 pins. 5 patients had infections of Checkett-Otterburn grade 2, altogether involving 4 hydroxyapatite-coated and 2 bisphosphonate-coated pins. In 1 case, the infection lasted for 2 weeks; the others resolved within a week. Pain estimation at removal was median 4 for the bisphosphonate-coated pins and 5 for the hydroxyapatite-coated pins. The difference was –1 unit (range 2 to –6).

The difference between metaphyseal removal and insertion torque was 2.4 Nm (SD 1.0) for the bisphosphonate pins and it was 2.3 Nm (SD 1.6) for the hydroxyapatite-coated pins (difference = 0.08, CI: –0.68 to 0.84).

Removal torque for the metaphyseal pins was 4.4 Nm (SD 1.3) for the bisphosphonate-coated pins and 4.2 Nm (SD 1.6) for the hydroxyapatite-coated pins (difference = 0.2, CI: –0.5 to 1.0).

## Discussion

The bisphosphonate-coated screws in the shaft showed a removal torque similar to that of uncoated controls, and those in the metaphysis were similar to the hydroxyapatite-coated ones. The confidence intervals exclude any important differences. The problems with loosening in the metaphysis—which have precluded the use of uncoated screws—were absent.

The primary hypothesis was that a bisphosphonate coating would improve fixation in the shaft. We failed to show this. The hypothesis was based on the previous observation that pins in the shaft have lower removal torque than insertion torque (Toksvig-Larsen and W-Dahl 2008). This was thought to be a result of peri-implant resorption, which we hoped to inhibit with the coating. In this study, no deterioration of fixation occurred in the controls, and there was therefore nothing for the bisphosphonate to inhibit. A previous (similar) study with systemic bisphosphonate treatment (1 injection of zoledronate) showed a higher extraction moment in the bisphosphonate group (Harding et al. 2010). Again, the controls showed lower values than in the present study. We have no explanation for the better fixation of the uncoated pins compared to the previous studies.

None of the metaphyseal pins loosened. This can be seen against the background of a previous study of similar design involving 19 patients, where all 19 uncoated metaphyseal pins had gross loosening (Magyar et al.1997). Considering these poor results with uncoated metaphyseal pins, we considered it unethical to compare the bisphosphonate-coated pins with uncoated, even though this would have been ideal to demonstrate the effect of the coating. It should be acknowledged that the trial was not designed as a non-inferiority trial for the metaphyseal pins, since this was a secondary goal.

It is our impression that external fixation with threaded pins has become less popular in recent years, possibly because of perceived poor fixation over time in metaphyseal bone. This problem might be avoided by use of a bisphosphonate coating.

The metaphyseal pins in this study were used for weight bearing without restriction, and could carry considerable load for months without loosening. It is not certain that bisphosphonate-coated pins can do that on their own, as each patient also had one hydroxypatite pin. However, because there were only 2 metaphyseal pins, it is likely that the forces were rather evenly distributed between them. Moreover, in the previous study comparing uncoated and hydroxyapatite-coated pins, the uncoated pins loosened in spite of the fact that they also worked in pair with a coated pin.

Our results are in line with those in a recent study of dental implants (Abtahi et al. 2012a). In that study, patients also received paired implants, 1 of which was coated with bisphosphonate with a similar but not identical method (the coating procedure has been simplified for the current study). The outcome variable was based on measurement of the resonance frequency of vibrations applied to the exposed implant at insertion and after 6 months. The resonance frequency is a result of the mechanical stiffness of the construct of the implant and the surrounding bone, and is therefore seen as a proxy for fixation. There was a clinically important increase in resonance frequency with the bisphosphonate coating. The findings from dental implants and now from external fixation pins together suggest that the principle of applying a bisphosphonate coating to metal might be useful for many types of implants. There is a concern that inhibited resorption around a bisphosphonate-coated implant could aggravate a possible infection, as removal of infected bone might be an important part of the defense. So far, we have not seen this, either in this study or in the dental study. Indeed, we have shown the contrary in an experimental model, where a bisphosphonate coating improved fixation and allowed healing in spite of bacterial contamination. This effect may be related to the very localized bisphosphonate application, because systemic bisphosphonate treatment was found to be deleterious in the same setting (Abtahi et al. 2012b).

In conclusion, the present study suggests that bisphosphonate-coated external fixation pins can be used successfully in metaphyseal bone, where uncoated pins have a history of showing a very high rate of loosening.
